# eHealth Literacy and Beliefs About Medicines Among Taiwanese College Students: Cross-sectional Study

**DOI:** 10.2196/24144

**Published:** 2021-11-30

**Authors:** Chiao Ling Huang, Chia-Hsun Chiang, Shu Ching Yang

**Affiliations:** 1 Faculty of Education Department of Educational Information Technology East China Normal University Shanghai China; 2 Intelligent Electronic Commerce Research Center Institute of Education National Sun Yat-Sen University Kaohsiung Taiwan

**Keywords:** beliefs about medicines, college student, eHealth literacy, health education

## Abstract

**Background:**

Good eHealth literacy and correct beliefs about medicines are beneficial for making good health care decisions and may further influence an individual's quality of life. However, few studies have discussed these two factors simultaneously. Moreover, gender differences are associated with health literacy and beliefs about medicines. Therefore, it is important to examine the multiple relationships between college students’ eHealth literacy and beliefs about medicines, as well as gender differences.

**Objective:**

This study aims to (1) examine the multiple relationships between eHealth literacy and beliefs about medicines and (2) analyze gender differences in eHealth literacy and beliefs about medicines with Taiwanese college students.

**Methods:**

We used a paper-and-pencil questionnaire that included age, gender, 3-level eHealth literacy, and beliefs about medicines to collect data. In total, 475 data points were obtained and analyzed through independent *t* tests and canonical correlation analyses.

**Results:**

The *t* test (*t*_473_*=*3.73; *P*<.001; *t*_473_=–2.10; *P*=.04) showed that women had lower functional eHealth literacy and more specific concerns about medicines than men. Canonical correlation analyses indicated that the first and second canonical correlation coefficients between eHealth literacy and beliefs about medicines reached a significant level, implying that a multivariate relationship indeed existed.

**Conclusions:**

These findings reveal that women in Taiwan have lower functional eHealth literacy and stronger concerns about medicines than men. In addition, students with higher eHealth literacy have more positive perceptions of and beliefs about medicines.

## Introduction

Beliefs about medicines refer to a concept related to the cognitive representation of medicines. Such beliefs are used to evaluate individuals’ beliefs regarding the necessity of and problems associated with prescribed medication and their more general beliefs about the overuse and detrimental effects of medicines [1]. According to the theory of self-regulation, beliefs about medicine are related to the decision to take medicine [1]. Studies have shown that nonadherence to medication regimens is more likely to occur among those with negative views regarding their medication, and that these views are accompanied by stronger concerns about potential harm, which caused them to believe that taking medicines is harmful in general [2,3].Therefore, helping people accurately understand medicines’ use can be a key element in reducing misunderstandings regarding medication and further building positive attitudes toward medication.

In addition to directly help people establish correct notions of medication use, enhancing individuals’ health literacy may be another useful approach because the process of forming beliefs about medicines may depend on information seeking and processing. Health literacy is the capability of individuals to access, comprehend, and effectively utilize health-related information, and it is a critical factor in the disease management and health promotion arena [4]. When patients request more information and written information is clear and easily understood, patients are assisted in improving their knowledge of and adherence to treatment [5]. Additionally, studies have shown that patients with poor health literacy are more inclined to incorrectly interpret labels and health information [6], more often possess negative emotionality that affect adherence [7], are more likely to believe that medications are necessary, and are more concerned about the possible side effects of their medications [8]. Namely, individuals with poor health literacy are less likely to comprehend information about diseases and medicines and have negative beliefs about medicines.

The internet has become a popular way to search for, obtain, and share health-related information over the last decade. Nevertheless, the abilities needed to collect and evaluate information through the internet differ from those needed to use books and other hard copies. In the cyberworld, individuals must be equipped with the ability, resources, and motive to seek, understand, and evaluate web-based health information [9]. eHealth literacy, which consists of functional, interactive, and critical levels [10], is defined as the capability to acquire needed and correct health information through the internet and further use this information to resolve health questions or make health decisions [11]. Apparently, the need for eHealth literacy is becoming increasingly important, as it is an indicator of how an individual applies web-based health information in his/her life [12]. Past studies have found that individuals with limited functional eHealth literacy may consult doctors frequently and that those with higher critical eHealth literacy can more effectively utilize health services [13]. However, research clarifying the multivariate correlations between eHealth literacy and beliefs about medicines is relatively scant. Furthermore, most previous studies on beliefs about medicines have focused on chronic disease patients [1,3,8,14] rather than normal samples, creating an academic gap.

In Taiwan, one can quickly and conveniently find information about medicinal drugs and disorders via the internet [15]. Before starting college, students' health literacy and beliefs about medicines are mostly influenced by parents and teachers. However, autonomous learning is encouraged in higher education. In this learning atmosphere, students have more opportunities to seek information on the internet and develop strong independent thinking and self-care abilities. Therefore, eHealth literacy and beliefs about medicines that students develop during college may well influence whether they will make good health care decisions in adulthood. Moreover, gender may play different roles in health literacy and beliefs about medicines. Females are reportedly more likely to have lower health literacy [16-18], stronger concerns about medicines [14], firmer beliefs about the necessity of taking medicine [19], and firmer beliefs that medications are overprescribed [20] than males.

Therefore, to obtain a comprehensive understanding of this topic and to address the lack of other samples in previous studies, with a focus on health education in Taiwan, the present study aims to examine the multiple relationships between college students’ eHealth literacy and beliefs about medicines and to analyze gender differences. Specifically, we attempted to answer the following questions: What is the relationship between eHealth literacy and beliefs about medicines among college students? Further, what is the effect or role of gender in these two concepts? Based on the extant literature, we propose the following hypotheses:

1. Taiwanese college students with higher eHealth literacy are more likely to have positive perceptions of and beliefs about medicines.
2. Taiwanese women are more likely to have lower eHealth literacy and stronger beliefs about medicines than men.

## Methods

### Study Design and Participants

We collected 2 samples from Taiwan; 1 sample was used for pretesting, and the other sample was used for the formal study. Pretesting was employed to validate the appropriateness of the research instrument used in this study by performing an exploratory factor analysis (EFA) to confirm the construct validity. The formal study was used to analyze the relation between college students’ eHealth literacy and their beliefs about medicines and the associated gender differences.

During pretesting, a convenience sampling approach was used to recruit students to participate in this investigation. We contacted our acquaintances who teach at other universities to help us promote this research and distribute the questionnaires. In total, 199 data points were returned and analyzed in the pretest study. A stratified random sampling method was adopted for the formal study. Specifically, we divided Taiwan into 3 regions and then used a computer to randomly select schools. For the selected schools, we contacted their instructors and asked whether they were willing to assist with distributing the questionnaire. Ultimately, 500 questionnaires were distributed, and 475 were returned. Among the 475 responses, missing values for each question did not exceed 2. We used the series mean to replace these missing data.

### Measures

#### eHealth Literacy Scale

The participants’ eHealth literacy was measured by the eHealth Literacy Scale (eHLS) [21]. The eHLS measures functional (3 items; eg, “I find the online health information difficult to understand”), interactive (4 items; eg, “I can locate health information efficiently through search engines”), and critical literacy (5 items; eg, “I will think about whether the online health information applies to my situation”). Functional eHealth literacy refers to basic competency in reading and writing web-based health information. Interactive eHealth literacy refers to the communication and social competencies used to consume information in a web-based social multimedia environment. Critical eHealth literacy involves people's cognitive competency in appraising, judging, or evaluating web-based information relevant to health [21]. According to Chiang et al’s [21] report, item analysis, EFA, and confirmatory factor analysis (CFA) were employed to determine the reliability and validity of eHLS. Specifically, the results of item analysis revealed that the comparisons between extreme measures ranged from 3.93 to 7.31 (*P*<.001), and the coefficient of correlation ranged from 0.70 to 0.85 (*P*<.01). The EFA and CFA results revealed that the Kaiser–Meyer–Olkin (KMO) measure was 0.83, the Bartlett test for sphericity was significant (*P*<.001), the factor loadings ranged from 0.53 to 0.90, the explained variance was 61.10%, the standardized factor loadings ranged from 0.60 to 0.86 (*P*<.001), composite reliability ranged from 0.75 to 0.84, and the average variance extracted for each dimension ranged from 0.50 to 0.52. In addition, for the goodness-of-fit indexes, *χ*^2^_51_=139.00, comparative fit index=0.96, root mean square error of approximation=0.06, and standardized root mean square residual=0.05. Each item in the eHLS was rated by the respondents on a 5-point Likert scale, with 1 indicating strong disagreement and 5 indicating strong agreement. Higher eHLS scores indicated that the participants had higher eHealth literacy, and all the variables were regarded as continuous variables. According to Sharma’s [22] guidelines, the reliability of a Likert-type rating scale can be obtained by computing the value of Cronbach α. The Cronbach α values obtained in our study sample were .82 (functional), .83 (interactive) and .87 (critical).

#### Beliefs About Medicines Scale

The Beliefs About Medicines Scale (BMS) was designed by the authors on the basis of Horne et al’s [1] scale. Three specialist professors in this field helped test the content validity of the BMS. We provided each professor with two sheets: one contains clear information about this scale, including this measurement’s purpose and each dimension’s definition, and the other contains an evaluation form with 2 options (inappropriate and appropriate) for each item. Experts were asked to complete the evaluation form and suggest modifications for items they rated inappropriate. Based on their suggestions, we revised and confirmed the BMS items until all experts were satisfied. The BMS includes specific and general sections measuring college students’ beliefs about medicines (Multimedia Appendix 1). The respondents rated each item in the BMS on a 5-point Likert scale, where 1 indicated strong disagreement and 5 indicated strong agreement, and all the variables were regarded as continuous variables. The results of the EFA (principal axis factors method with direct oblimin rotation) resulted in a 4-factor structure, which is the same as Horne et al’s [1] scale, and revealed that the factor loadings ranged from 0.54 to 0.88 and that the explained variance was 59.12%. Before conducting the EFA, we examined the results of the KMO and Bartlett sphericity tests to ensure that these data were appropriate for performing an EFA. According to Sharma’s [22] guidelines, the reliability of a Likert-type rating scale can be obtained by computing the value of Cronbach α. The Cronbach α values obtained in our study sample were .77 (specific-necessity, 4 items; eg, “I cannot live without my medicines”), .76 (specific-concern, 4 items; eg, “Taking medicines worries me”), .72 (general-overuse, 3 items; eg, “Most medicines are addictive”), and .84 (general-harm, 2 items; eg, “Doctors prescribe too many medicines”), respectively.

### Data Analysis

In the pretest study, peer review was employed to test the content validity of the 13-item BMS, and an EFA was used to assess its construct validity. In the formal study, we calculated the internal consistency coefficients (α values) of each instrument and performed a descriptive statistical analysis, independent *t* tests, and a canonical correlation analysis to gain a better understanding of our samples and clarify the relationship among the research variables. Notably, all statistical analyses were performed using SPSS. A value of *P*<.05 was considered statistically significant.

### Ethical Considerations

This study used an anonymous questionnaire to gather data, which is consistent with the government’s institutional review board rules for exempt review. All participation was voluntary and confidential. To ensure anonymity and confidentiality, we asked the students not to write any personal information on the questionnaire, and all the questionnaires were stored in a locked cabinet that only the researchers could access. Before distributing the questionnaires, the lecturer clearly informed the participants of the study aim and noted that they had the right to refuse to participate at any time without penalty; in addition, the participants were informed that their participation would not have any influence on their grades.

## Results

### Descriptive Statistics of eHealth Literacy and Beliefs About Medicines

Most of the students were under 22 years of age, except for 31 students whose ages ranged from 23 to 40 years, and the average age of our participants was 20.37 years. Table 1 presents the descriptive statistics of eHealth literacy and beliefs about medicines, showing that the college students basically had a moderate or high self-perceived level of eHealth literacy (all the means exceed 3). The participants also had low perceptions of the necessity of medicines (mean 1.78), low concerns about medications (mean 2.88), and low levels of the belief that medicines are harmful (mean 2.44) and overused (mean 2.50).

**Table 1:** Descriptive analysis of eHealth literacy and beliefs about medicines.

Attribute			Value, mean (SD)
**eHealth literacy**			
	Functional	3.95 (0.77)	
	Interactive	3.73 (0.70)	
	Critical	3.81 (0.72)	
**Beliefs about medicines**			
	Specific-necessity	1.78 (0.72)	
	Specific-concerns	2.88 (0.94)	
	General-harm	2.44 (0.83)	
	General-overuse	2.50 (0.94)	

### Gender Differences in eHealth Literacy and Beliefs About Medicines

The *t* test results shown in Table 2 reveal that men had higher functional eHealth literacy than women (mean_men_ 4.07, mean_women_ 3.81; *t*_473_=3.73; *P*<.001) and that women had stronger concerns about medications than men (mean_men_ 2.79, mean_women_ 2.97; *t*_473_=–2.10; *P*=.04).

**Table 2:** *t* test for gender differences in eHealth literacy and beliefs about medicines.

Attributes		Score (men; n=250), mean (SD)	Score (women; n=225), mean (SD)	*t* test (*df*)	*P* value
**eHealth literacy**					
	Functional	4.07 (0.77)	3.81 (0.75)	3.73 (473)	<.001
	Interactive	3.77 (0.69)	3.69 (0.72)	1.22 (473)	.22
	Critical	3.84 (0.72)	3.77 (0.72)	1.07 (473)	.29
**Beliefs about medicines**					
	Specific-necessity	1.74 (0.72)	1.82 (0.71)	–1.10 (473)	.27
	Specific-concerns	2.79 (0.93)	2.97 (0.95)	–2.10 (473)	.04
	General-harm	2.40 (0.84)	2.47 (0.82)	–0.92 (473)	.36
	General-overuse	2.46 (0.95)	2.55 (0.94)	–1.07 (473)	.28

### Relationship Between eHealth Literacy and Beliefs About Medicines

The results of the canonical correlation analysis presented in Table 3 reveal that the first and second canonical correlation coefficients between eHealth literacy and beliefs about medicines were 0.28 and 0.15, respectively, which reached a significant level (*P*<.05).

The results of the first canonical variate indicated that the students with relatively high functional and interactive eHealth literacy were less concerned about the 4 aspects related to the medicines used. The results of the second canonical variate revealed that the students with relatively high critical eHealth literacy were less concerned about the specific necessity of the medicines used.

**Table 3:** Canonical correlation analysis of eHealth literacy and beliefs about medicines.

Attributes			First canonical variate		Second canonical variate
**eHealth literacy set, *r_s_*^a^**					
	Functional	*0.96*		–0.17	
	Interactive	*0.50*		0.07	
	Critical	0.48		*0.74*	
	Variance percentage	46.94		19.46	
	Redundancy	3.65		0.44	
**Beliefs about medicines set, *r*_s_**					
	Specific-necessity	–*0.63*		–*0.69*	
	Specific-concerns	–*0.63*		0.34	
	General-harm	–*0.77*		0.37	
	General-overuse	–*0.60*		–0.07	
	Variance percentage	43.42		18.25	
	Redundancy	3.38		0.42	
R_c_^b^			0.28		0.15
R_c_^2^			0.08		0.02
*P* value			<.001		.03

^a^*r*_s_: structure coefficients (canonical loadings) and absolute values of ≥0.50 are italicized.

^b^R_c_: canonical correlation coefficients.

## Discussion

### Principal Findings

This study found that women had lower functional eHealth literacy and higher specific concern about medicines than men; hence, hypothesis 2 is only partially supported by our findings. Previous studies have found that female sex is associated with limited functional health literacy [16,17]. Researchers have argued that the lower level of health literacy among females was probably owing to their low educational level [17,18]. However, this study found that despite similar education levels, functional eHealth literacy may also have gender differences.

Functional literacy is closely linked to reading comprehension and numeracy skills [23]. Although reading comprehension competence is similar between the sexes [24], studies have found that females have poorer numeracy skills than males [25], including those with higher education [26]. Poorer numeracy may account for the phenomenon that female students were more likely to have inadequate functional eHealth literacy than male students, but this merits further study. In addition, overestimation and underestimation of eHealth literacy may have occurred because self-reporting was used for assessment. It is possible that functional eHealth literacy is similar between male and female college students. Future studies could develop a direct test of eHealth literacy and clarify whether a difference in eHealth literacy exists on the basis of gender among college students.

Consistent with Viktil et al [14], this study found that women worried more about the potential negative effects of medication than men. Physiological sex differences may result in a dissimilar incidence rate of disease and response to drug therapy [27]. Studies have shown that females are more likely to use gender-specific drugs and general medications [28] and have more adverse drug reactions than males [28,29]. In addition, it is worth noting that women’s adverse drug reactions might have been ignored in pharmaceutical research for many years. In contrast, men’s adverse drug reactions have gained more attention. For example, independent safety committees ceased Behre et al’s [30] study because the committees found that the injectable combination hormonal contraceptive for men had some side effects (ie, depression and mood changes) and the risks outweighed the potential benefits for the participants. Thus, women's concerns about medication are not simply owing to the physiological differences between the 2 sexes but also because pharmaceutical research has ignored the side effects of medication for women (or, in the absence of relevant experimental data, it is unclear what side effects these drugs have on women). In this case, if women need to take medication for treatment, they have no other choice but to adapt to the current medication, which may elicit women's concerns. Therefore, this finding’s result should be interpreted with caution.

Hypothesis 1 is also only partially supported by the results of the canonical correlation analysis. The first canonical result indicated that students with relatively high functional eHealth literacy were less concerned about the 4 aspects related to medicine. According to Taiwan’s Ministry of Health and Welfare, relevant indications should be printed on medication packaging, such as drug names, cautions, principal indications, and main adverse reactions [15]. Functional health literacy particularly emphasizes basic reading skills, which are used to address health information [10,31,32]. Lower functional health literacy is a barrier preventing patients from understanding their health conditions, such as diseases and proper treatment [33]. Previous studies have also shown that individuals with poor functional health literacy tend to have the attitude that drug therapy is requisite and are inclined to express concerns about the possible adverse reactions or sequela of their medications [8].

Therefore, compared to students with higher functional eHealth literacy, those with lower functional eHealth literacy might have poorer abilities to understand information related to their medications and thus believe that they need more personal medication and have more concerns about the potential negative effects of medicines; such students were also more inclined to believe that medicines are harmful and overused by physicians. Scholars have argued that illustrated medication information provides useful reinforcement [34], and for individuals with low health literacy, illustrations enhance their health knowledge and adherence to medications [35]. Thus, illustrated medication information could help students with low eHealth literacy build positive perceptions and beliefs about medicines.

Moreover, the results of the first set of canonical correlations showed that students with higher interactive eHealth literacy tended to have positive perceptions and beliefs about medicines. In Taiwan, people can conveniently and quickly search for information about medications and disorders through the internet, news, and magazines [15]. Interactive eHealth literacy identifies the communication and social competency that are employed in consuming health information in the web-based environment [13,21,36]. Students with adequate interactive eHealth literacy have the capability to collect information and extract meaning from various types of communications and further build positive beliefs about medicines. A study found that a summary information leaflet, regular health presentations and monthly meetings can effectively change patients’ health education concept and positively influence their attitudes toward medicines [37]. Therefore, health education practitioners could conduct regular health education campaigns to build positive beliefs about medicines for students with low interactive eHealth literacy.

Finally, the results of the second set of canonical correlation analysis revealed that students with adequate critical eHealth literacy were less likely to have stronger perceptions of a need for medications to maintain their present and future health. Critical eHealth literacy refers to the most advanced cognitive competency that is used to critically appraise and evaluate web-based information relevant to health [13,21,36]. Given that web-based health information often lacks evidence and is incorrect or misinforming, patients are easily confused and likely to form inaccurate and negative beliefs about medications [38]. Cultivating students’ critical health literacy is necessary because critical literacy can help them analyze health information and take an active role in addressing their health-related issues [39], thus giving college students lower perceptions of the need for medications to maintain their current and future health.

### Limitations of This Study

Although this study contributes to our understanding of the relevant correlates of eHealth literacy and beliefs about medicines, it has some limitations. First, this study used a cross-sectional design. Thus, we gathered data at a single time point and could not determine the development of beliefs about medicines along with eHealth literacy. Second, since the 2 scales (eHLS and BMS) applied in this study did not have predictive validity information, these scales’ utility may be questionable. We suggest that future studies should consider this issue, using more complete scales to address this concern. However, even with this flaw, the study provides a good starting point for further studies in general. Third, our participants may have been likely to overestimate or underestimate their eHealth literacy and beliefs about medicines owing to social desirability expectations. Future studies could adopt research methods other than self-reporting assessment to address this issue. Fourth, the sample in the current study was restricted by age to college students in Taiwan. The findings should be interpreted considering the sample’s homogeneity. In particular, students’ different demographic characteristics (eg, major, type of family, religious practices, and community) may influence beliefs about medicines and eHealth literacy. Future studies can use diverse samples and perform covariance analysis to control for these potentially influential factors. Finally, students’ healthy skepticism of medications is not documented as part of this study. Healthy skepticism helps people wary of medicine information and gauges medications’ value from a different perspective. Future studies could consider this variable.

### Conclusions

To the best of our knowledge, this study is the first to explore the relationship among the 3 levels of eHealth literacy and 4 aspects of beliefs about medicines among college students, especially Taiwanese college students. Our study contributes not only to research but also to educational practice. Our results indicate that higher the eHealth literacy of Taiwanese college students, the more positive the perceptions and beliefs about medicines they held, thus providing some insights for health educational practitioners who could help college students build positive beliefs about medicines by promoting their eHealth literacy. In addition, this study found that women had lower functional eHealth literacy and stronger concerns about medicines than men. Therefore, health educators could develop gender-specific programs to improve women’s functional eHealth literacy and reduce their concerns about medicines.

## Appendix

Multimedia Appendix 1 Each section of the Beliefs About Medicines Scale.

## Abbreviations

BMS: Beliefs About Medicines Scale
CFA: confirmatory factor analysis
EFA: exploratory factor analysis
eHLS: eHealth Literacy Scale
KMO: Kaiser–Meyer–Olkin
