# Supplementary material for: eHealth Literacy and Beliefs About Medicines Among Taiwanese College Students: Cross-sectional Study
Source: JMIR Med Inform. 2021 Nov 30;9(11):e24144. doi: 10.2196/24144 (PMC8672294; doi:10.2196/24144)
Supplement: Multimedia Appendix 1 [file medinform_v9i11e24144_app1.docx]

| Aspect | | Purpose and scoring |
| --- | --- | --- |
| **Specific section:**  Measuring individuals’ beliefs about the medications used for specific illnesses | | |
| 1 | specific-necessity  (four items) | Evaluating personal beliefs about the necessity of taking medicine. Higher scores indicate stronger views of a personal need for medication to maintain current and future health. |
| 2 | specific-concern  (four items) | Regarding concerns about medications prescribed to control an illness. Higher scores indicate stronger concerns regarding the side effects of medications. |
| **General section:**  Measuring general beliefs about medicine overuse and harm. Higher scores indicate that the participants had higher beliefs that medicines in general are overused by physicians and are harmful. | | |
| 1 | general-overuse  (three items) | Regarding views of how medicines are used by physicians. |
| 2 | general-harm  (two items) | Regarding beliefs about the degree to which patients perceive medicines as essentially harmful. |
